# A Fire Detection Method Based on a Mind-Linked Continuous-Coupled Neural Network

**DOI:** 10.3390/biomimetics11060410

**Published:** 2026-06-10

**Authors:** Kangrong Liu, Ji Wang, Wei Yang, Shiwei Wang, Jianxiang Wang, Jinhai Zhang, Zhaorui Zhang, Xinlei An, Jizhao Liu

**Affiliations:** 1 School of Information Science and Engineering, Lanzhou University, Lanzhou 730000, China; lkangrong2023@lzu.edu.cn (K.L.); liujz@lzu.edu.cn (J.L.); 2 National Key Laboratory on Vacuum Technology and Physics, Lanzhou Institute of Physics, Lanzhou 730000, China; 3 School of Mathematics and Physics, Lanzhou Jiaotong University, Lanzhou 730070, China; 4 School of Electronics and Communication Engineering, Lanzhou University of Arts and Sciences, Lanzhou 730010, China

**Keywords:** ML-CCNN, brain-inspired computing, fire detection, smoke alarm

## Abstract

With the development of Internet of Things (IoT) technology, fire detection systems based on multi-sensor fusion have become critical infrastructure to ensure public safety. Due to environmental noise and sensor heterogeneity, these systems often suffer from high rates of false alarms and missed detections. Although existing machine learning approaches have partially improved classification accuracy, their overall performance remains limited. Inspired by the cognitive mechanisms of the human brain, we developed an improved mind-linked continuous-coupled neural network (ML-CCNN) based on the existing continuous-coupled neural network (CCNN). We propose a parameter adaptation mechanism that modulates neural activations through a global threshold. We utilized the synthetic minority oversampling technique (SMOTE) to mitigate data imbalance and transformed sample feature vectors into matrices for training. Our model achieved an accuracy of 99.96% on our own dataset and 99.97% on the public Smoke Detection Dataset (SDD), which highlights ML-CCNN’s potential for fire detection.

## 1. Introduction

Fire is a sudden and destructive disaster that poses a serious threat to human life and property. Statistical evidence indicates that fire-related damage is generally inversely proportional to the timeliness of its detection [1]. Consequently, the accuracy and response speed of fire detection technologies are critical for disaster prevention. Traditional fire detection systems primarily rely on threshold-based decisions using a single physical parameter, such as smoke or temperature [2]. However, real-world scenarios are subject to various sources of interference, including dust accumulation, steam generated during cooking, and fluctuations in carbon dioxide concentrations in crowded environments. These sudden environmental variations may trigger false alarms [3].

With the increasing integration of sensor technology and the IoT, multi-sensor fire detection has become a promising approach. This method detects fires by simultaneously acquiring multi-dimensional data, including temperature, humidity, CO_2_, and particulate matter concentrations (PM1.0 and PM2.5). Traditional machine learning approaches, such as support vector machines (SVMs), logistic regression (LR), and naive bayes (NB) classifiers, detect fires by constructing classification models [3,4]. However, their performance is limited by the complex characteristics of fire data, which restricts their effectiveness in practical fire detection tasks [2]. HDBMS algorithm [5] that dynamically selects the best-performing machine learning model based on real-time data to further improve prediction accuracy. Latest deep learning models, such as EIF-LSTM [6] and BiLSTM-LN-SA [7], have also achieved certain results by improving long short-term memory (LSTM) networks. Building on these advances, we propose a brain-inspired computing model for fire detection.

Neuroscience has long been an essential driver of progress in artificial intelligence (AI) [8]. Related models such as spiking neural networks (SNNs) [9] and pulse coupled neural networks (PCNNs) [10] have advanced rapidly in recent years. As a novel brain-inspired computing model, the continuous-coupled neural network (CCNN) introduces a probabilistic activation mechanism to generate continuous outputs, addressing the limitations of traditional spiking models [11]. However, some parameters in the CCNN model are manually predefined, which limits the adaptability of the model to complex datasets. Therefore, it is necessary to further improve the generalization ability of CCNN while preserving its brain-inspired mechanism.

The rest of this paper is organized as follows. Section 2 reviews the related work. Section 3 introduces the proposed model. Section 4 presents the experiments. Section 5 concludes this paper.

## 2. Related Work

Currently, the main approaches to fire detection can be categorized into two groups: computer vision-based detection methods and deep learning-based IoT detection methods. In contrast, this study proposes a novel detection approach based on brain-inspired computing.

### 2.1. Computer Vision-Based Fire Detection

Vision-based methods primarily utilize images or videos captured by cameras to detect fires by analyzing the visual features of flames and smoke [12,13,14,15]. Early video-based fire detection methods heavily relied on features such as color and texture [16]. However, these methods exhibited poor generalization capabilities in complex scenarios [13]. To address these limitations, CNN-based vision methods have gradually become the dominant approach. Muhammad et al. [17] proposed a lightweight CNN architecture based on MobileNet for resource-constrained surveillance networks. Their study showed that lightweight CNN architectures can reduce computational cost while maintaining high fire classification accuracy.

In addition to image classification, real-time object detection algorithms have been widely used for fire detection. Li and Zhao [18] investigated several CNN-based object detection methods for image-based fire detection and showed that the YOLOv3-based method achieved higher average precision among the compared models. Subsequently, Sun et al. [19] proposed an improved YOLOv5 method for forest fire monitoring by incorporating the convolutional block attention module (CBAM), a small target detection layer, and the Ghost module, thereby improving detection performance under complex background conditions.

However, visual detection methods have inherent limitations. These methods are highly sensitive to variations in environmental illumination and may fail to detect obscured fire sources.

### 2.2. Deep Learning-Based Multi-Sensor Fire Detection

Multi-sensor fire detection systems typically integrate sensors for temperature, humidity, smoke concentration, and carbon monoxide (CO), enabling fire detection using multi-dimensional data fusion. Nakip et al. [20] proposed a recurrent trend predictive neural network (rTPNN) for multi-sensor fire detection. Their method is based on both trend and level prediction and the fusion of sensor readings and captures trends in multivariate time-series data collected using a multi-sensor detector. Deng et al. [21] further proposed an indoor fire detection method based on multi-sensor fusion and a lightweight convolutional neural network (CNN) for resource-constrained embedded platforms. Liu et al. [6] proposed a model named EIF-LSTM that uses LSTM networks to process continuous time-series sensor readings to extract temporal characteristics of environmental information. By increasing the sources and dimensions of information fusion, the detection accuracy of EIF-LSTM on the training set and the test set exceeds 96.5%. He et al. [7] proposed a novel model named BiLSTM-LN-SA to enhance robustness and accuracy. The model integrates a bidirectional long short-term memory (BiLSTM) network with layer normalization (LN) and a self-attention (SA) mechanism. Extensive evaluation using a real-world dataset demonstrates the superiority of the BiLSTM-LN-SA model, achieving a test accuracy of 98.38%. Although time-series models such as LSTM have achieved relatively high accuracy, they still suffer from limited sensitivity to weak fire signals under complex environmental interference.

### 2.3. Brain-Inspired Computing

The human brain is composed of billions of neurons that accomplish information transmission and processing through spikes. SNN as a promising brain-inspired computational model with binary spike information transmission mechanism, rich spatially temporal dynamics, and event-driven characteristics [22]. As a result, they have attracted extensive attention in recent years and have gradually been applied to fields such as pattern recognition, image processing, and intelligent perception [23,24,25,26]. Among various brain-inspired computing models, the PCNN employs a modulated coupling mechanism to modulate key parameters, while the coupling results produce internal activity [27]. The PCNN was first proposed by Eckhorn et al. [28] based on the phenomenon of synchronized oscillations in the cat visual cortex. Its core idea is to simulate the synchronous pulse-firing mechanism of the biological visual cortex by utilizing neuronal coupling and dynamic threshold mechanisms. Owing to its characteristics of synchronous oscillation, pulse coupling, and training-free operation, the PCNN has been widely studied and applied in tasks such as image segmentation, image fusion, object detection, and feature extraction [28,29,30].

With the development of brain-inspired visual models, various improved structures have been further proposed based on the PCNN, among which the continuous-coupled neural network (CCNN) enhances the representation of complex temporal and spatial patterns through continuous neuron activation and dynamic coupling mechanisms [31,32,33]. The CCNN introduces a probabilistic activation mechanism and exhibits dynamics that are more consistent with those of real neurons [11]. However, existing CCNN models still rely on manually predefined static parameters [30]. This limits their adaptability in fire detection scenarios characterized by complex backgrounds and significant noise interference. To address this issue, this study proposes an improved ML-CCNN model based on the CCNN. By introducing a learnable global threshold, the proposed model enhances its adaptive capability in complex environments.

## 3. Fire Detection Framework Based on the ML-CCNN Model

### 3.1. CCNN Neuron

The CCNN is inspired by the dynamics of primary visual cortex, exhibits commensurate static and dynamic properties with real neurons [33]. It uses sigmoid functions to replace the pulse generator and exhibits highly complex chaotic behavior under periodic stimulation. In contrast to SNNs and PCNNs [34], the CCNN exhibits periodic behavior under DC stimulation and chaotic behavior under AC stimulation [11].

The mechanism of the CCNN is described using a set of recursive equations. The state of each CCNN neuron is primarily determined by five modules: feeding input, couple linking, modulation product, dynamic activity, and continuous output [11]. The formulas are as follows:

(1) $$F_{ij}\left(n\right)=e^{-\alpha_{f}}F_{ij}\left(n-1\right)+V_{F}M_{ijkl}*Y_{kl}\left(n-1\right)+S_{ij},$$

(2) $$L_{ij}\left(n\right)=e^{-\alpha_{l}}L_{ij}\left(n-1\right)+V_{L}W_{ijkl}*Y_{kl}\left(n-1\right),$$

(3) $$U_{ij}\left(n\right)=F_{ij}\left(n\right)\left(1+\beta L_{ij}\left(n\right)\right),$$

(4) $$Y_{ij}\left(n\right)=sigmoid\left(U_{ij}\left(n\right)-E_{ij}\left(n\right)\right),$$

(5) $$E_{ij}\left(n\right)=e^{-\alpha_{e}}E_{ij}\left(n-1\right)+V_{E}Y_{ij}\left(n-1\right),$$

The notations are explained in Table 1.

At each time step, the CCNN neuron updates its feeding input $F_{ij}$ by considering the exponentially decayed previous input, the weighted sum of neighboring neuron outputs, and the external stimulus. The couple linking $L_{ij}$ is calculated using the previous input with a decay factor and the corresponding weight matrix to capture interactions between neurons. The modulation product $U_{ij}$ combines $F_{ij}$ and $L_{ij}$, where the linking strength is $1+\beta L_{ij}$. The continuous output $Y_{ij}$ is then generated using a sigmoid activation function. Meanwhile, the dynamic activity $E_{ij}$ evolves over time by incorporating previous output values and an exponential decay term, allowing the neuron to adjust its refractory period dynamically.

### 3.2. The ML-CCNN Model for Fire Detection

The ML-CCNN model adopts the global threshold γ as a control parameter; the model can automatically calculate the dynamic activity and other internal hyperparameters. The parameter γ is implemented as a learnable parameter optimized together with other network parameters through backpropagation and ranges from 0.01 to 0.99. The mathematical expressions are as follows:

The decay factor α is defined as follows:

(6) $$\alpha =\ln\frac{1}{\gamma },$$

The linking strength β is defined as follows:

(7) $$\beta =e^{-\alpha },$$

The amplitude parameter $V_{E}$ in the dynamic activity is defined as follows:

(8) $$V_{E}=e^{-2\alpha }+e^{-3\alpha },$$

The decay factors for the feeding input ($\alpha_{f}$), the couple linking ($\alpha_{l}$), and the dynamic activity ($\alpha_{e}$) are defined as follows:

(9) $$\alpha_{f}=3\alpha ,$$

(10) $$\alpha_{l}=\alpha ,$$

(11) $$\alpha_{e}=\alpha ,$$

Based on the work of Yi et al. [35], $V_{E}$ and $V_{L}$ are defined as follows:

(12) $$V_{F}=1.0,$$

(13) $$V_{L}=1.0,$$

**Figure 1:**
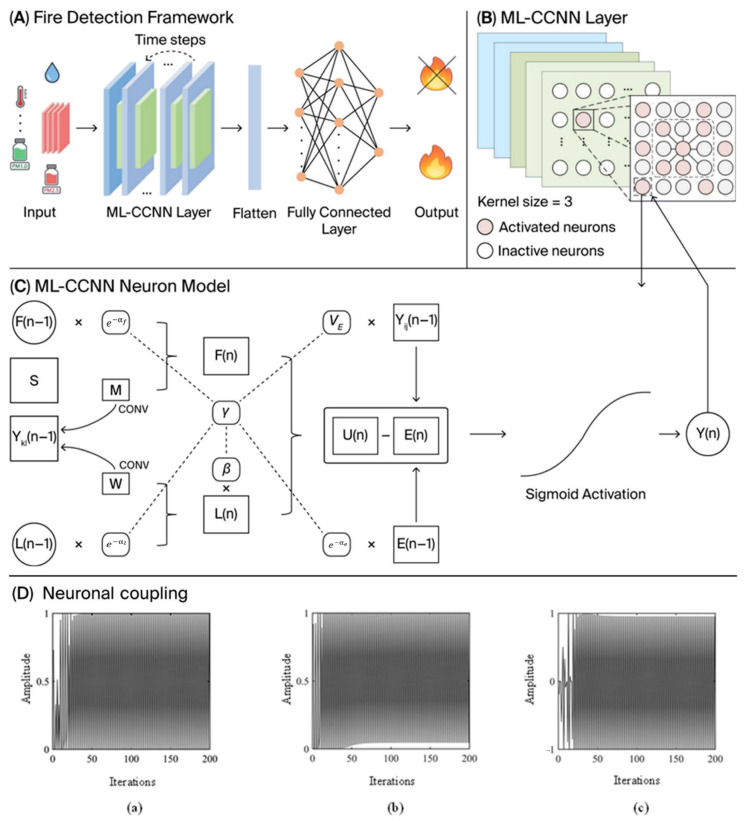
The ML-CCNN framework for fire detection: (**A**) the fire detection framework; (**B**) the ML-CCNN layer; (**C**) the ML-CCNN neuron model; (**D**) Neuronal coupling: (**a**) waveform of neuron 1 with the stimulation intensity of S=1; (**b**) waveform of neuron 2 with the stimulation intensity of S=2; (**c**) Difference in neuron 1 and neuron 2.

Figure 1 shows the ML-CCNN model for fire detection. Because visual networks depend on multi-dimensional matrix inputs, they exhibit a dimensional mismatch with conventional datasets formatted as one-dimensional feature vectors. To address this, this study employs a data format conversion scheme that populates the elements of the feature vector into a two-dimensional matrix (N×N), with any remaining positions filled with zeros. This process re-encodes the one-dimensional samples into single-channel feature maps, enabling the visual model to perform feature extraction.

The model uses two connection matrixes, *W* and *M*, with bias-free convolution operations. The model enables the precise capture of spatial details in the input samples while extracting the temporal evolution features of fire signals. Consequently, this enhances the model’s ability to capture sample information in complex environments.

The model first calculates the decay factor α, the linking strength β, and the amplitude parameter $V_{E}$. Based on the decay factor α, the decay factors for the feeding input $\alpha_{f}$, the couple linking $\alpha_{l}$, and the dynamic activity $\alpha_{e}$ are calculated using Equations (9)–(11). For each neuron, the feeding input F(0), the couple linking L(0), and the continuous output Y(0) are initialized to zero, whereas the initial dynamic activity E(0) is set to $\frac{V_{E}}{e^{-\alpha }}$. During the T time steps, $F_{ij}\left(n\right)$ and $L_{ij}\left(n\right)$ are updated using Equations (1) and (2), and the final output $Y_{ij}\left(n\right)$ is then calculated using Equations (3)–(5).

The ML-CCNN layer iteration process is shown in Algorithm 1.
**Algorithm 1** ML-CCNN layer iteration algorithm.**1: Input:** $X=\left[X^{1},X^{2},\ldots ,X^{T}\right]$, Parameters γ, W, M **2: Output:** $y_{mlccnn}$ **3:** Create an empty list $Y_{seq}=\left[\;\right]$ **4:** Calculate $\alpha =\ln\left(1/\gamma \right)$ **5:** Initialize states: F(0) = 0, L(0) = 0, Y(0) = 0 **6:** Initialize threshold: $E\left(0\right)=\frac{V_{E}}{e^{-\alpha }}$ **7: for**
t=1, 2, …, T **do** **8:**         $F\left(t\right)=\mathrm{BN}\left(e^{-3\alpha }F\left(t-1\right)+M*Y\left(t-1\right)+X\left(t\right)\right)$ **9:**         $L\left(t\right)=\mathrm{BN}\left(e^{-\alpha }L\left(t-1\right)+W*Y\left(t-1\right)\right)$ **10:**       $U\left(t\right)=F\left(t\right)\cdot \left(1+e^{-\alpha }L\left(t\right)\right)$ **11:**       $E\left(t\right)=e^{-\alpha }E\left(t-1\right)+V_{E}Y\left(t-1\right)$ **12:**       $Y\left(t\right)=\sigma \left(U\left(t\right)-E\left(t\right)\right)$ **13:**      Append Y(t) to $Y_{seq}$ **14: end for** **15:** Stack $Y_{seq}$ to obtain $y_{ml-ccnn}$ **16: return**
$y_{ml-ccnn}$

The ML-CCNN layer iterates over the input sequence *X* for *T* time steps, updating the states of each neuron according to the values from the previous time step and the current input data. After the T-step iterations, the output $y_{ml-ccnn}$ is generated. The $y_{ml-ccnn}$ is then flattened and fed into the fully connected layer. The final output is given as follows:

(14) $$y_{out}=A_{2}\cdot \left(\mathrm{ReLU}\left(A_{1}\cdot y_{ml-ccnn}+c_{1}\right)\right)+c_{2},$$

where $A_{1}$ and $c_{1}$ denote the weight and bias of the first fully connected layer, respectively, whereas $A_{2}$ and $c_{2}$ represent those of the output layer. In addition, the ReLU activation function provides the nonlinear activation for the network [36] and is defined as follows:

(15) $$ReLU\left(x\right)=max\left(0,x\right),$$

During parameter optimization, the model utilizes a cross-entropy loss function that measures the difference between the predicted probability distribution of the model and true probability distribution of the labels [37]. The cross-entropy loss function is defined as follows:

(16) $$loss=-\frac{1}{N}\sum_{i=1}^{N}\sum_{c=1}^{C}y_{ic}log\left(p_{ic}\right),$$

where N represents the total number of samples in a batch, and C denotes the number of classes in the classification task. Here, $y_{ic}$ is the one-hot encoded ground-truth label, and $p_{ic}$ represents the predicted probability that sample i belongs to class c. The probability $p_{ic}$ is calculated as follows:

(17) $$p_{ic}=\arg\underset{c}{\max}\hat{y_{\mathrm{out}}},$$

where $\hat{y_{\mathrm{out}}}$ denotes the output score of the fully connected layer for sample i and class c.

In addition, the adaptive moment estimation (Adam) optimizer is employed for parameter optimization, with the initial learning rate set to $\eta =10^{-3}$.

## 4. Experiments

In this section, we describe the data preprocessing procedure, the evaluation metrics, and the analysis of the performance of the proposed fire detection framework.

### 4.1. Dataset

Using a standard smoke detector tunnel, we constructed a smoke alarm dataset named the Fire Alarm Dataset (FAD). The FAD contains 21,856 samples collected under the combustion conditions of wood, sponge, and n-heptane. This dataset includes 18,342 normal samples (83.92% of the total samples) and 3514 fire samples (16.08% of the total samples) (see Table 2).

**Figure 2:**
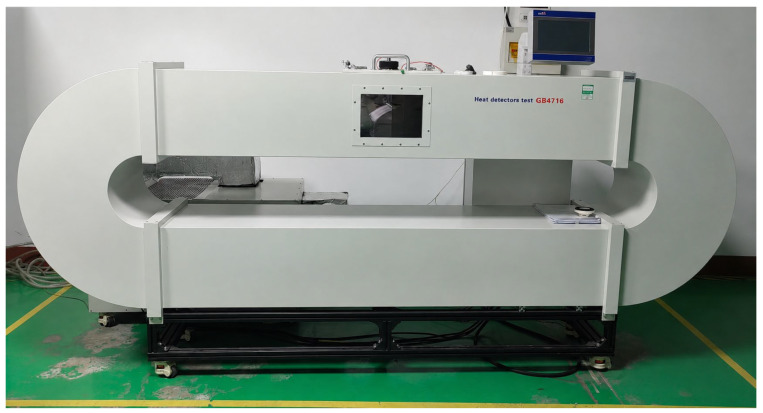
ZB-SMK-III standard smoke detector tunnel.

The ZB-SMK-III standard smoke detector tunnel is shown in Figure 2. The integrated sensor included an ionization sensor (ION) and a light obscuration rate sensor (LOR), both for smoke detection. In addition, three temperature sensors (Temp1–Temp3) were used to record temperatures at different positions, and the reading from thermostat (Temp4) was used as an additional temperature measurement. Furthermore, three humidity sensors (Humi1–Humi3) were used to measure air humidity. The label denotes the environmental status, where 0 indicates a normal condition and 1 indicates a fire condition. The dataset features are shown in Table 3.

Additionally, the public Smoke Detection Dataset (SDD) [38] from Kaggle was also used. The dataset contains 62,630 samples, including 44,757 fire samples (71.46% of the total samples) and 17,873 normal samples (28.54% of the total samples). The dataset features are shown in Table 4.

### 4.2. Data Preprocessing

Due to the class imbalance between normal and fire samples in the datasets, we used the synthetic minority over-sampling technique (SMOTE) proposed by Chawla et al. [39]. SMOTE is a method used to generate minority class samples based on a geometric interpolation mechanism in the feature space to balance the dataset. This approach effectively alleviates the class imbalance problem.

SMOTE balances the dataset by synthesizing new minority samples in the feature space [39]. The specific generation process consists of three main steps. First, a target sample is randomly selected from the minority class set. Second, several K-nearest neighbors of this sample are found in the feature space. Finally, new minority samples are synthesized using random linear interpolation on the line segment between the target sample and a random neighbor sample [39]. The pseudo-code for SMOTE is shown in Algorithm 2.
**Algorithm 2** SMOTE AlgorithmDataset $D=\{\left(x_{i},y_{i}\right){\}}_{i=1}^{N}$, where $x_{i}=\left(LON,LOR,\ldots ,Humi2,Humi3\right)$ and $y_{i}\in \{0,1\}$. Minority class samples $D_{\mathrm{mnrt}}$, majority class samples $D_{\mathrm{mjrt}}$. Number of synthetic samples to generate $N_{\mathrm{synthetic}}$, number of nearest neighbors k. **Ensure:** Augmented dataset $D_{augmented}$ 1: Initialize $D_{augmented}\leftarrow D$ 2: Extract minority class features: $X_{\mathrm{minority}}=\{x_{i}|y_{i}=1\}$ 3: Compute k nearest neighbors for each sample $x\in X_{minority}$ using Euclidean distance in the feature space 4: **for** i = 1 **to**  $N_{\mathrm{synthetic}}$
**do** 5:            Randomly select a minority class sample $x_{i}\in X_{minority}$ 6:            Randomly select one of its k-nearest neighbors $x_{nn}$ 7:            Generate a random interpolation factor $\lambda ∼U\left(0,1\right)$ 8:            Calculate a synthetic sample:$x_{\mathrm{new}}=x_{i}+\lambda \cdot \left(x_{nn}-x_{i}\right)$9:            Append $x_{\mathrm{new}}$ to $X_{minority}$ 10: **end for** 11: Combine augmented minority class samples with majority class samples:$X_{augmented}=\left(x,1\right)|x\in X_{\mathrm{minority}}\cup X_{\mathrm{majority}}$12: **return**  $X_{augmented}$

**Figure 3:**
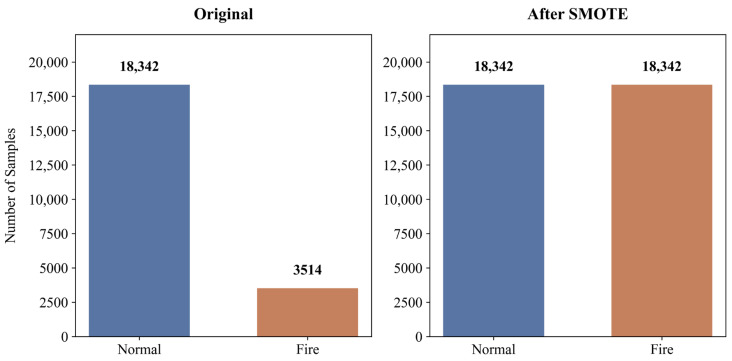
Comparison of the FAD before and after applying SMOTE.

As shown in Figure 3, after applying SMOTE, the number of fire samples in the FAD increased from 3514 to 18,342. Meanwhile, the number of normal samples remained unchanged at 18,342.

**Figure 4:**
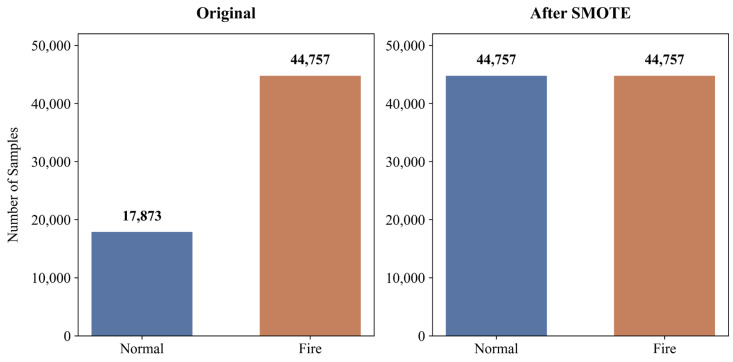
Comparison of the SDD before and after applying SMOTE.

As shown in Figure 4, after applying SMOTE, the number of normal samples in the SDD increased from 17,873 to 44,757, thereby achieving class balance. Meanwhile, the number of fire samples remained unchanged at 44,757.

The SMOTE-processed datasets were divided into training and testing sets at a 7:3 ratio [40]. The resulting sample distributions for the FAD and SDD are presented in Table 5.

### 4.3. Data Input

To enable the ML-CCNN to effectively process one-dimensional features, the original feature vectors were transformed into a single-channel matrix. This transformation allows the model to exploit convolutional operations for feature extraction and adapt to the input format required by visual model [31]. Specifically, the features of the FAD and SDD were filled into a matrix in a left-to-right and top-to-bottom order. The FAD features were arranged into a 3 × 3 matrix, whereas the SDD features were arranged into a 4 × 4 matrix. Any remaining positions in these matrixes were padded with zeros. The resulting matrixes are denoted as follows:

(18) $$M=\left[\begin{array}{ccc} Ion & LOR & Temp1\\ Temp2 & Temp3 & Temp4\\ Humi1 & Humi2 & Humi3 \end{array}\right]$$

(19) $$M=\left[\begin{array}{cccc} Temperature & Humidity & TVOC & eCO2\\ RawH2 & RawEthanol & Pressure & PM1.0\\ PM2.5 & NC0.5 & NC1.0 & NC2.5\\ 0 & 0 & 0 & 0 \end{array}\right]$$

Following this transformation, the original one-dimensional data are reconstructed into single-channel images, thereby adapting to the input requirements of the ML-CCNN.

### 4.4. Performance Metrics

To comprehensively evaluate the performance of the model in the fire detection task, we employ four standard evaluation metrics: accuracy, precision, recall, and F1-score.

Accuracy reflects the proportion of correct predictions made by the model across the entire dataset, representing the ratio of correctly predicted samples to the total number of samples [41]. It is defined as follows:

(20) $$\mathrm{Accuracy}=\frac{TP+TN}{TP+TN+FP+FN}$$

In high-risk scenarios such as fire detection, the consequence of missed detections is highly severe. Therefore, precision and recall are introduced to further evaluate the model’s performance. Precision measures the proportion of actual fire samples among all samples predicted as fire by the model [42] and is defined as follows:

(21) $$\mathrm{Precision}=\frac{TP}{TP+FP}$$

Recall represents the probability that actual fire samples are correctly detected [42] and is defined as follows:

(22) $$\mathrm{Recall}=\frac{TP}{TP+FN}$$

To comprehensively evaluate the balance between precision and recall, the F1-score is adopted as the harmonic mean of the two metrics [42]. It is calculated as follows:

(23) $$F1\text{-}Score=2\times \frac{\mathrm{Precision}\times \mathrm{Recall}}{\mathrm{Precision}+\mathrm{Recall}}$$

### 4.5. Experimental Results

**Figure 5:**
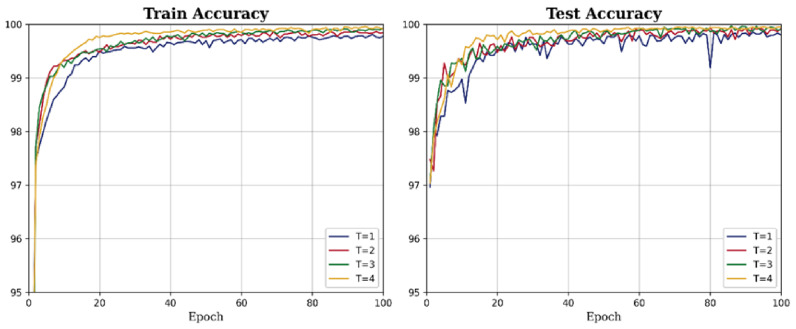
Training results of the ML-CCNN on the FAD.

**Figure 6:**
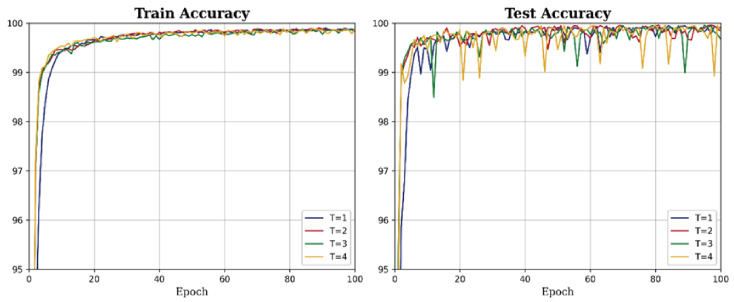
Training results of the ML-CCNN on the SDD.

The model was trained for 100 epochs, with the training loss and testing accuracy recorded at each epoch. Figure 5 and Figure 6 show the performance of the model on the FAD and SDD, respectively, with the time step T set to 1, 2, 3, and 4. Table 6 shows the training parameters.

As shown in Figure 5 and Figure 6, the ML-CCNN exhibited rapid convergence under all tested time steps. On the FAD, the training accuracy increased quickly during the early epochs and gradually stabilized near 100%, indicating that the model was able to fit the training data effectively. The model trained on the SDD also converged rapidly. The training accuracy remained stable after several training epochs. Although the testing accuracy showed slightly larger fluctuations than that on the FAD, especially at time steps T = 3 and T = 4, its overall trend remained stable and close to 100%.

To evaluate the proposed model, we compared it with several conventional machine learning models, including support vector machine (SVM), logistic regression (LR) and naive Bayes (NB). These conventional models are directly available in Python’s scikit-learn (v1.5.1) libraries. We also included spiking neural networks (SNNs) and the continuous-coupled neural network (CCNN) as brain-inspired baseline models. All models were trained and evaluated on the FAD and SDD using the same data preprocessing strategy with SMOTE.

**Figure 7:**
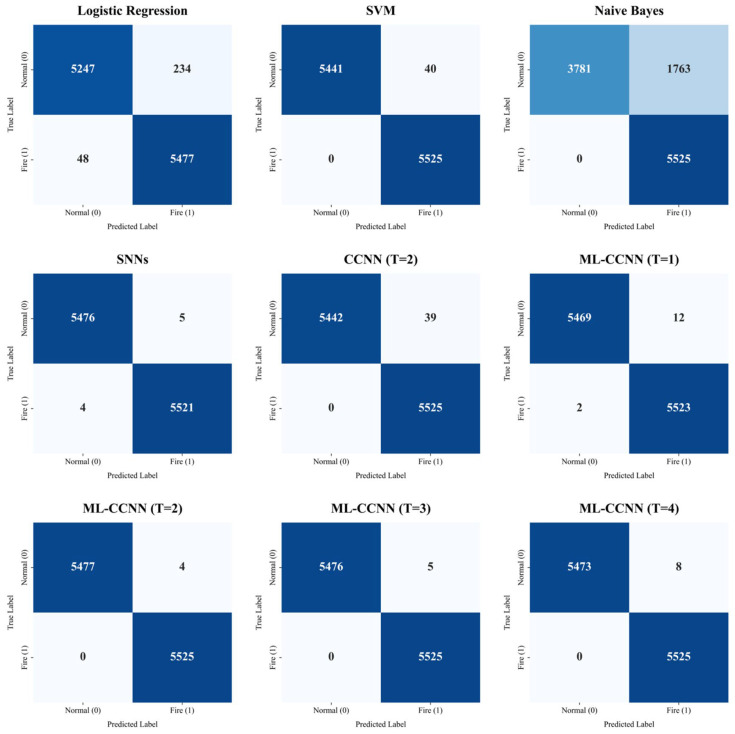
Confusion matrix of the results on the FAD.

Table 7 presents the performance of the models trained on the FAD, while Figure 7 shows the confusion matrix.

**Figure 8:**
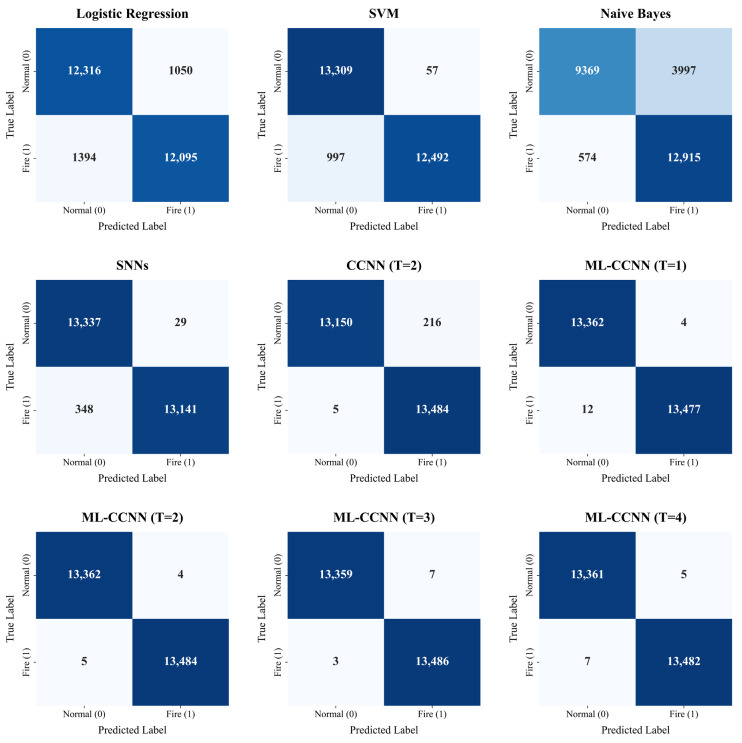
Confusion matrix of the results on the SDD dataset.

Table 8 presents the performance comparison results of the models trained on the SDD, while Figure 8 shows the confusion matrix.

Based on the results presented in Table 6 and Table 7, the ML-CCNN demonstrated stable classification performance on both the FAD and SDD under different time-step settings. When the time step was set to T = 2, the model achieved optimal performance. On the FAD, the ML-CCNN (T = 2) achieved an accuracy of 0.9996, precision of 0.9993, recall of 1.0000, and F1-score of 0.9996, indicating that the model could classify almost all samples correctly. When the time step increased to T = 3 and T = 4, the performance of the model showed a slight decline.

On the SDD, the ML-CCNN also maintained outstanding performance under different time-step settings. The model achieved an accuracy of 0.9997, precision of 0.9997, recall of 0.9996, and F1-score of 0.9997, achieving the optimal performance among the four configurations when T = 2. Although ML-CCNN (T = 3) reached the highest recall of 0.9998, its accuracy and F1-score were both slightly lower than those of ML-CCNN (T = 2). These results indicated that the ML-CCNN could effectively capture complex features in multi-sensor data and maintain stable fire detection performance.

Among conventional models, SVM achieved an accuracy of 0.9964 and precision of 0.9928 on the FAD. By comparison, LR achieved an accuracy of 0.9744 and precision of 0.9590, whereas NB performed relatively poorly, with an accuracy of 0.8398 and precision of 0.7581. Although NB attained a high recall of 1.0000 its F1-score was only 0.8624. For the SDD, the conventional models showed a more evident performance gap. SVM achieved an accuracy of 0.9608 and F1-score of 0.9595, whereas LR and NB performed comparatively poorly, with accuracies of 0.9090 and 0.8298. Overall, these results indicated that conventional machine learning models showed limited adaptability.

The brain-inspired baseline models were also used for comparison. On the FAD, SNNs obtained an accuracy of 0.9992 and an F1-score of 0.9992, while CCNN (T = 2) achieved an accuracy of 0.9965 and an F1-score of 0.9965. Although CCNN (T = 2) reached a recall of 1.0000, its precision decreased to 0.9930 due to the increased number of false positives. On the SDD, SNNs achieved an accuracy of 0.9860 and an F1-score of 0.9859, and CCNN (T = 2) achieved better overall performance, with an accuracy of 0.9918 and an F1-score of 0.9919. The accuracy of SNNs decreased slightly, and CCNN (T = 2) still produced more false positives, resulting in a lower precision of 0.9842. These results show that SNNs and CCNN can capture fire features, but their accuracy is lower than that of the proposed ML-CCNN.

In summary, experimental results demonstrate that ML-CCNN shows effective capability in extracting complex features from multi-sensor data. At T = 2, the model performs better than other models, demonstrating its superiority in fire detection tasks.

### 4.6. Cross-Validation

To comprehensively evaluate the generalization capability of the model and verify its performance stability across different data subsets, we performed ten-fold cross-validation on the FAD and SDD. Each fold was trained for 100 epochs to ensure model convergence.

As shown in Table 9 and Table 10, the validation results of ML-CCNN remained stable across all folds on both the FAD and SDD. Specifically, on the FAD (Table 8), the validation accuracy ranged from 0.9989 to 0.9997, with an average of 0.9993. On the SDD (Table 9), the validation accuracy varied between 0.9951 and 0.9999, with an average of 0.9987. These validation results indicate that the ML-CCNN model achieved stable performance across different variation folds.

### 4.7. Comparison with Other Fire Detection Models

We retrieved recent studies on fire detection, as summarized in Table 11. The methods in these studies cover a range of approaches, including traditional machine learning and deep learning techniques. The proposed model achieves an accuracy of 99.96%, indicating competitive performance in fire detection tasks.

### 4.8. γ Sensitivity Analysis

As shown in Table 12 and Table 13, we tested the model on FAD and SDD with γ initialized to 0.05, 0.1, 0.3, 0.5, 0.7, and 0.9. Performance remained stable across different γ values on the FAD. On the SDD, performance decreased slightly with increasing γ. Overall, the model demonstrates high robustness to the initialization of γ, and relatively low to moderate initial values are more favorable.

### 4.9. Ablation Study

Ablation experiments were conducted to evaluate the contribution of different components in the ML-CCNN. As shown in Table 14 and Table 15, the complete ML-CCNN achieves the best performance on both FAD and SDD. Removing *M*, *W*, $V_{E}$, coupling modulation, or dynamic activity leads to different degrees of performance degradation. Although the differences in accuracy and F1-score are relatively small, the increased false positives and false negatives indicate that these components help reduce false alarms and missed alarms. Overall, the results demonstrate that the proposed components jointly improve the robustness of the ML-CCNN.

## 5. Conclusions

In this work, we proposed a fire detection framework based on the ML-CCNN. The model introduces a parameter adaptation mechanism utilizing a global threshold parameter that dynamically regulates key parameters to enhance the representation capability of the network. Combined with the SMOTE method for dataset balancing and a vector-to-matrix transformation strategy, the model achieves 99.96% accuracy on the FAD and 99.97% on the SDD. Experimental results show that the proposed model achieves superior performance in terms of accuracy compared with other models, demonstrating its strong competitiveness for fire detection.

## Figures and Tables

**Table 1:** Explanations of the notations used in the CCNN neuron model.

Symbol	Explanation
$\alpha_{f},\alpha_{l},\alpha_{e}$	Decay factors for the feeding input, linking input, and dynamic activity, respectively, which record previous neuronal input states.
$V_{F},V_{L}$	Weighting factors modulating the action potentials of surrounding neurons.
β	Linking strength that directly determines $L_{ij}$ in the modulation product $U_{ij}.$
$F_{ij}$	Feeding input, reflecting the current signal received by the neuron.
$S_{ij}$	External feeding input received by the receptive fields.
$L_{ij}$	Couple input, representing the interactions between neurons.
$U_{ij}$	Modulation product determined by both the feeding input and the coupling connection input.
$Y_{ij}$	Continuous output calculated using the sigmoid activation function.
$E_{ij}$	Dynamic activity, regulating the refractory period of the neuron and cooperating with the continuous output function.
$M_{ijkl},W_{ijkl}$	Weight matrices that define the strength of signal transmission from neighboring neurons to the current neuron in the feeding input and couple input. Their values are automatically adjusted during training.
*	Convolution operation, representing the interaction between the weight matrix and the output signal from previous time steps, signals from adjacent neurons in the neighborhood converge onto the current neuron.
sigmoid	Activation function that converts $U_{ij}(n)-E_{ij}(n)$ into a continuous output between 0 and 1.

**Table 2:** Class distribution in the FAD.

Class	Number of Samples	Percentage
Normal	18,342	83.92%
Fire	3514	16.08%
Total	21,856	100%

**Table 3:** Features of the FAD.

Feature	Description
ION	Reading from the ionization sensor
LOR	Light obscuration rate
Temp1	Temperature recorded by the first temperature sensor
Temp2	Temperature recorded by the second temperature sensor
Temp3	Temperature recorded by the third temperature sensor
Temp4	Reading from the thermostat
Humi1	Humidity recorded by the first humidity sensor
Humi2	Humidity recorded by the second humidity sensor
Humi3	Humidity recorded by the third humidity sensor
label	Sample label, where 0 denotes a normal condition and 1 represents a fire condition

**Table 4:** Features of the SDD.

Feature	Description
Temperature	Ambient temperature measured in degrees Celsius
Humidity	Relative ambient humidity percentage
TVOC	Total volatile organic compound concentration
eCO_2_	Equivalent CO_2_ concentration
Raw H2	Raw hydrogen gas signal output
Raw Ethanol	Raw ethanol gas signal output
Pressure	Ambient air pressure
PM1.0	Particulate matter concentration (diameter < 1.0 μm)
PM2.5	Particulate matter concentration (diameter < 2.5 μm)
NC0.5	Number concentration of particles (diameter < 0.5 μm)
NC1.0	Number concentration of particles (diameter < 1.0 μm)
NC2.5	Number concentration of particles (diameter < 2.5 μm)
Fire Alarm	Sample label, where 0 denotes a normal condition and 1 represents a fire condition

**Table 5:** Sample distribution of the FAD and SDD after splitting.

Dataset	Class	Training	Test
FAD	Normal	12,861	5481
	Fire	12,817	5525
SDD	Normal	31,391	13,366
	Fire	31,268	13,489

**Table 6:** Training parameters.

Parameter	Setting
Learning rate	0.001
Batch size	64
Epochs	100
Train/test split	70%/30%
Initial γ	0.05
Loss function	Cross-entropy loss
Kernel size	3 × 3
Time step T	1, 2, 3, and 4

**Table 7:** Comparison of model performance on the FAD.

Model	TP	FP	TN	FN	Accuracy	Precision	Recall	F1-Score
Logistic Regression	5477	234	5247	48	0.9744	0.9590	0.9913	0.9749
SVM	5525	40	5441	0	0.9964	0.9928	1.0000	0.9964
Naïve Bayes	5525	1763	3718	0	0.8398	0.7581	1.0000	0.8624
SNNs	5521	5	5476	4	0.9992	0.9991	0.9993	0.9992
CCNN (T = 2)	5525	39	5442	0	0.9965	0.9930	1.0000	0.9965
ML-CCNN (T = 1)	5523	12	5469	2	0.9987	0.9978	0.9996	0.9987
ML-CCNN (T = 2)	5525	4	5477	0	0.9996	0.9993	1.0000	0.9996
ML-CCNN (T = 3)	5525	5	5476	0	0.9995	0.9991	1.0000	0.9995
ML-CCNN (T = 4)	5525	8	5473	0	0.9993	0.9986	1.0000	0.9994

**Table 8:** Comparison of model performance on the SDD.

Model	TP	FP	TN	FN	Accuracy	Precision	Recall	F1-Score
Logistic Regression	12,095	1050	12,316	1394	0.9090	0.9201	0.8967	0.9082
SVM	12,492	57	13,309	997	0.9608	0.9955	0.9261	0.9595
Naïve Bayes	12,915	3997	9369	574	0.8298	0.7637	0.9574	0.8496
SNNs	13,141	29	13,337	348	0.9860	0.9978	0.9742	0.9859
CCNN (T = 2)	13,484	216	13,150	5	0.9918	0.9842	0.9996	0.9919
ML-CCNN (T = 1)	13,477	4	13,362	12	0.9994	0.9997	0.9991	0.9994
ML-CCNN (T = 2)	13,484	4	13,362	5	0.9997	0.9997	0.9996	0.9997
ML-CCNN (T = 3)	13,486	7	13,359	3	0.9996	0.9995	0.9998	0.9996
ML-CCNN (T = 4)	13,482	5	13,361	7	0.9996	0.9996	0.9995	0.9996

**Table 9:** Ten-fold cross-validation results on the FAD.

Fold	TP	FP	TN	FN	Accuracy	Precision	Recall	F1-Score
1	1825	2	1841	1	0.9992	0.9989	0.9995	0.9992
2	1860	2	1807	0	0.9995	0.9989	1.0000	0.9995
3	1840	2	1827	0	0.9995	0.9989	1.0000	0.9995
4	1856	3	1810	0	0.9992	0.9984	1.0000	0.9992
5	1804	2	1862	0	0.9995	0.9989	1.0000	0.9994
6	1876	4	1788	0	0.9989	0.9979	1.0000	0.9989
7	1836	2	1830	0	0.9995	0.9989	1.0000	0.9995
8	1809	2	1857	0	0.9995	0.9989	1.0000	0.9994
9	1780	0	1887	1	0.9997	1.0000	0.9994	0.9997
10	1854	4	1810	0	0.9989	0.9978	1.0000	0.9989
Mean					0.9993	0.9988	0.9999	0.9993

**Table 10:** Ten-fold cross-validation results on the SDD.

Fold	TP	FP	TN	FN	Accuracy	Precision	Recall	F1-Score
1	4537	8	4407	0	0.9991	0.9982	1.0000	0.9991
2	4462	33	4457	0	0.9963	0.9927	1.0000	0.9963
3	4447	1	4461	43	0.9951	0.9998	0.9904	0.9951
4	4365	3	4582	2	0.9994	0.9993	0.9995	0.9994
5	4522	0	4428	1	0.9999	1.0000	0.9998	0.9999
6	4481	1	4467	2	0.9997	0.9998	0.9996	0.9997
7	4481	2	4466	2	0.9996	0.9996	0.9996	0.9996
8	4438	7	4506	0	0.9992	0.9984	1.0000	0.9992
9	4438	2	4508	3	0.9994	0.9995	0.9993	0.9994
10	4531	1	4417	2	0.9997	0.9998	0.9996	0.9997
Mean					0.9987	0.9987	0.9988	0.9987

**Table 11:** Performance comparison with representative fire detection models.

Year	Venue	Model	Accuracy
2018	ICIT	FireDS-IoT (K-NN) [43]	0.9315
2018	ICIT	FireDS-IoT (Decision tree) [43]	0.8925
2019	IJSCAI	SVM [44]	0.8000
2020	IJACSA	GRU [45]	0.9989
2021	IEEE Access	rTPNN [20]	0.9600
2021	MDPI Information	BPNN [46]	0.9967
2022	ICAC3N	SVMs [47]	0.9750
2023	Sensors	ConvNeXt-FiRe [21]	0.9910
2023	IEEE ICET	CNN-BiLSTM-Attention [48]	0.9974
2023	NCA	EIF-LSTM [6]	0.9619
2025	Sensors	BiLSTM-LN-SA [7]	0.9838
		ML-CCNN(Ours)	0.9996

**Table 12:** Results of the γ sensitivity analysis on the FAD.

γ	TP	FP	TN	FN	Accuracy	Precision	Recall	F1-Score
0.05	5525	4	5477	0	0.9996	0.9993	1.0000	0.9996
0.1	5524	4	5477	1	0.9995	0.9993	0.9998	0.9995
0.3	5524	5	5476	1	0.9995	0.9991	0.9998	0.9995
0.5	5525	4	5477	0	0.9996	0.9993	1.0000	0.9996
0.7	5525	9	5472	0	0.9992	0.9984	1.0000	0.9992
0.9	5524	7	5474	1	0.9993	0.9987	0.9998	0.9993

**Table 13:** Results of the γ sensitivity analysis on the SDD.

γ	TP	FP	TN	FN	Accuracy	Precision	Recall	F1-Score
0.05	13,484	4	13,362	5	0.9997	0.9997	0.9996	0.9997
0.1	13,486	9	13,357	3	0.9996	0.9993	0.9998	0.9996
0.3	13,488	8	13,358	1	0.9997	0.9994	0.9999	0.9997
0.5	13,424	32	13,334	65	0.9964	0.9976	0.9952	0.9964
0.7	13,446	88	13,278	43	0.9951	0.9935	0.9968	0.9952
0.9	13,439	141	13,225	50	0.9929	0.9896	0.9963	0.9929

**Table 14:** Results of the ablation study on the FAD.

Model Variant	TP	FP	TN	FN	Accuracy	Precision	Recall	F1-Score
w/o *M*	5525	15	5466	0	0.9986	0.9973	1.0000	0.9986
w/o *W*	5525	8	5473	0	0.9993	0.9986	1.0000	0.9993
w/o $V_{E}$	5525	24	5457	0	0.9978	0.9957	1.0000	0.9978
w/o couple linking	5525	9	5472	0	0.9992	0.9984	1.0000	0.9992
w/o dynamic activity	5523	12	5469	2	0.9987	0.9978	0.9996	0.9987
ML-CCNN	5525	4	5477	0	0.9996	0.9993	1.0000	0.9996

**Table 15:** Results of the ablation study on the SDD.

Model Variant	TP	FP	TN	FN	Accuracy	Precision	Recall	F1-Score
w/o *M*	13,451	28	13,338	38	0.9975	0.9979	0.9972	0.9976
w/o *W*	13,453	23	13,343	36	0.9978	0.9983	0.9973	0.9978
w/o $V_{E}$	13,483	24	13,342	6	0.9989	0.9982	0.9996	0.9989
w/o couple linking	13,478	32	13,334	11	0.9984	0.9976	0.9992	0.9984
w/o dynamic activity	13,480	10	13,356	9	0.9993	0.9993	0.9993	0.9993
ML-CCNN	13,484	4	13,362	5	0.9997	0.9997	0.9996	0.9997

## Data Availability

The data presented in this study are openly available in Kaggle at https://www.kaggle.com/datasets/deepcontractor/smoke-detection-dataset/data (accessed on 22 February 2026).
